# A bibliometric analysis of literature on malaria vector resistance: (1996 – 2015)

**DOI:** 10.1186/s12992-016-0214-4

**Published:** 2016-11-25

**Authors:** Waleed M. Sweileh, Ansam F. Sawalha, Samah W. Al-Jabi, Sa’ed H. Zyoud, Naser Y. Shraim, Adham S. Abu-Taha

**Affiliations:** 1 Department of Physiology, Pharmacology, Toxicology, College of Medicine and Health Sciences, An-Najah National University, Nablus, 44839 Palestine; 2 Department of Clinical and Community Pharmacy, College of Medicine and Health Sciences, An-Najah National University, Nablus, 44839 Palestine; 3 Department of Pharmaceutical Chemistry and Technology, College of Medicine and Health Sciences, An-Najah National University, Nablus, 44839 Palestine

**Keywords:** Malaria, Vector, *Anopheles*, Resistance, Insecticide, Pyrethroids

## Abstract

**Background:**

Emergence of insecticide resistance in malaria vectors is a real threat to future goals of elimination and control of malaria. Therefore, the objective of this study was to assess research trend on insecticide resistance of *Anopheles* mosquito. In specific, number of publications, countries, institutions, and authors’ research profile, citation analysis, international collaborations, and impact of journals publishing documents on insecticide resistance will be presented. It was conducted via Scopus search engine which was used to retrieve relevant data. Keywords used were based on literature available on this topic. The duration of study was set from 1996–2015.

**Results:**

A total of 616 documents, mainly as original research articles (*n* = 569; 92.37%) were retrieved. The average number of citations per article was 26.36. Poisson log-linear regression analysis indicated that there was a 6.00% increase in the number of publications for each extra article on pyrethroid resistance. A total of 82 different countries and 1922 authors participated in publishing retrieved articles. The United Kingdom (UK) ranked first in number of publications followed by the United States of America (USA) and France. The top ten productive countries included seven African countries. The UK had collaborations mostly with Benin (relative link strength = 46). A total of 1817 institution/ organizations participated in the publication of retrieved articles. The most active institution/ organization was *Liverpool School of Tropical Medicine*. Retrieved articles were published in 134 different scientific peer reviewed journals. The journal that published most on this topic was *Malaria Journal* (*n* = 101; 16.4%). Four of the top active authors were from South Africa and two were from the UK. Three of the top ten cited articles were published in *Insect Molecular Biology* journal. Six articles were about pyrethroid resistance and at least two were about DDT resistance.

**Conclusion:**

Publications on insecticide resistance in malaria vector has gained momentum in the past decade. International collaborations enhanced the knowledge about the situation of vector resistance in countries with endemic malaria. Molecular biology of insecticide resistance is the key issue in understanding and overcoming this emerging problems.

## Background

The most common and dangerous malaria parasite, *Plasmodium falciparum* is transmitted by *Anopheles gambiae*. More than 100 species of *Anopheles* mosquito have been implicated in the transmission of human malaria. Prevention and reduction of malaria transmission requires control of *Anopheles* mosquito which can be done through insecticide – treated mosquito nets (ITNs) and indoor residual spraying (IRS) [1, 2]. The World Health Organization (WHO) approved the use of several insecticides for control of malaria vector. These insecticides belong to four chemical classes: organochlorines, organophosphates, carbamates andpyrethroids. For safety reasons, only pyrethroidsare suitable and safe for use in ITNs and long-lasting insecticidal nets (LLINs) while all approved insecticides are used and recommended for IRS [3–5].

The WHO is working toward an ambitious goal for the period 2016–2030 regarding control and elimination of malaria from different world regions [6], and future management of water resources in Africa, waste water irrigation, and water lifting devices [7–9]*.* The previous two decades had witnessed great achievements regarding malaria control [10, 11]. However, several challenges are facing the international agenda to control malaria by 2030. Most important challenges are the emergence of resistance to the new generation antimalarial drug resistance, artemisinins and the emergence of resistance among malaria vectors to common insecticides [12–19]. Insecticide resistance in malaria vectors is becoming a global concern because of reports on insecticide resistance from many countries in Africa [20–24]. Of particular concern is vector resistance to pyrethroids since they are the only class of insecticides approved for use in ITNs.

Research on insecticide resistance is highly needed in order to understand epidemiology and prevalence of the problem, the molecular mechanism of resistance, and to find strategies to overcome this problem. An initial step in understanding the size of the problem of insecticide resistance is analysis of available data and literature on this topic in order to assess what has been achieved. Actually, the intensive work of researchers in all fields of malaria has contributed to the control of this fatal infectious diseases. For example, Nobel Prize in 2015 was awarded for scientists who developed the artemisnins drugs which contributed significantly to malaria control [25–28].

Bibliometric analysis is a field in which several parameters and techniques are used in order to assess the volume, scientific impact, and trend of research on a particular topic. Bibliometric studies on insecticides in general have been carried out [29, 30]. Furthermore, bibliometric studies on malaria have also been published [31–35]. However, no biliometric studies have carried out on insecticide resistance of malaria vector. Therefore, the objective of this study was to assess and map research output on insecticide resistance of *Anopheles* mosquito, the malaria vector. In specific, the overall volume of publications, country, institution and author research profile on insecticides resistance will be presented. Furthermore, citation analysis, international collaborations, and impact of journals publishing on insecticide resistance of *Anopheles* mosquito will also be presented. Therefore, this study will be a new addition to the literature of malaria in general and insecticide resistance in malaria vectorsin particular. Research activity in vector-borne diseases is considered a priority for many world regions like Africa and South East Asia. Furthermore, research in vector-borne diseases must respond to the dynamic changes of these diseases and insecticide resistance is an emerging concern in this field in general and in malaria in particular. This was the major motive for this study.

## Methods

Scopus database was used to search for all published articles on insecticide resistance in malaria vector. Use of Scopus as a search engine was justifiable given the advantage that Scopus has over other databases [36]. Keywords used were those listed as insecticides for malaria vector control plus keywords pertaining to insecticides used in nets and residual spraying. These keywords were used in the title/ abstract fields. These keywords were used in combination with the word resistance or resistant. Retrieved documents were manually checked for validity of search strategy and articles that were outside the scope were deleted by adding the phrase “AND NOT” to the search query. For example, some of retrieved articles were about lice and scabies and had to be deleted. Manual validation was carried out by two of the co-authors who were aware of inclusion and exclusion criteria. Analysis of retrieved articles included listing bibliometric indicators mentioned in previous studies [37–49]. Analysis of results included regression models since this has been utilized successfully in previous studies [50–53]. Country, institution, source title, and authors were presented as top ten active items.

Collaboration between countries was presented as percentage of single country publication (SCP) and percentage of multiple country publication (MCP). The SCP represents intra-country collaboration while MCP represents inter country collaboration. Citation analysis for countries and journals was presented using VOSviewer technique [54]. The technique generates map that are either as density or network visualization maps. The map can be generated based on certain criteria set by the researchers. The impact of retrieved articles was assessed using Hirsch-index (*h*-index) [55]. The strength of journals publishing articles on malaria vector resistance was assessed by impact factor (IF) obtained from Journal Citation Report 2015.

## Results

### Publications’ numbers and citations

**Fig. 1:**
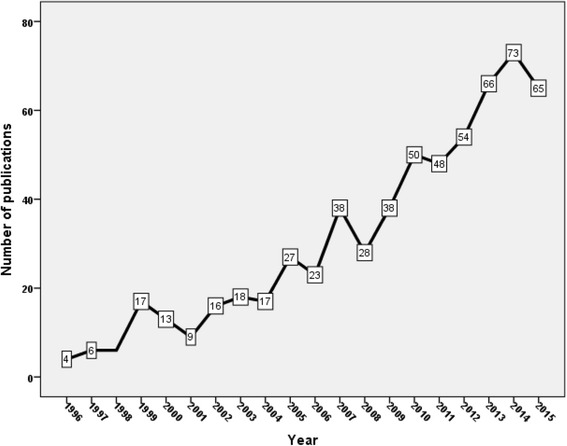
Growth of publications on malaria vector resistance to insecticides (1996–2015)

Results of the search query yielded a total of 616 documents, mainly as original research articles (*n* = 569; 92.37%). Most retrieved articles were in English (*n* = 588; 95.45%). The number of publications on insecticide resistance in malaria vector showed a dramatic growth in the past two decades (Fig. 1). The number of publications reached 73 documents in 2014 while there were only 4 documents published in 1996. The total number of citations of retrieved documents was 16238. Therefore, the average number of citations per article was 26.36. A total of 569 (92.37%) articles had at least one citation while 47 (7.63%) articles had no citations. The *h*-index of retrieved articles was 60. A scatter plot of the number of publications on vector resistance versus time yielded a straight line with a significant correlation and r value of 0.955. Poisson log-linear regression analysis indicated that publications on pyrethroid resistance is a significant predictor of worldwide research productivity on insecticide resistance in malaria vector. The model showed that the worldwide productivity will be 1.06 times greater for each extra article on pyrethroid resistance in malaria vector. In other words, there is a 6.00% increase in the number of publications for each extra article on pyrethroid resistance.

### Publications analysis based on authors and countries

**Fig. 2:**
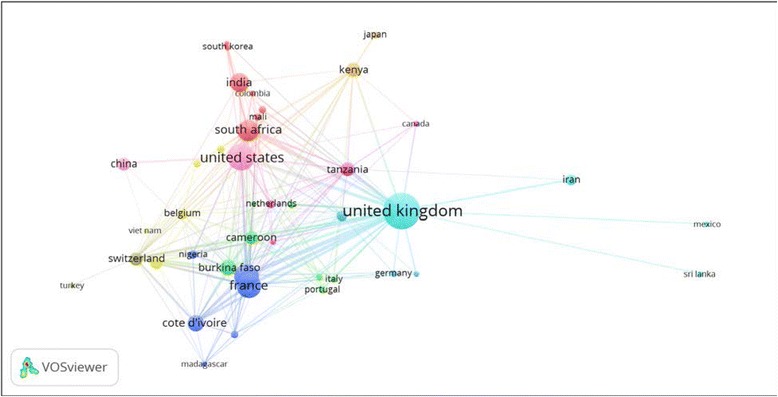
Network visualization map for country collaboration. A minimum of 5 documents per country was a set as threshold and 44 countries were included in the map. The thickness of the link between any two countries is indicative of extent of co-authorships (collaboration)

**Fig. 3:**
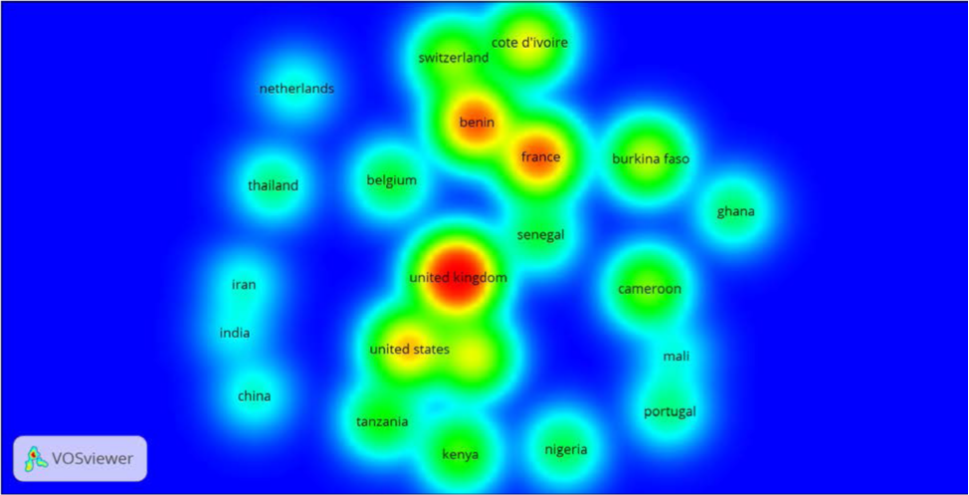
Density visualization map for country citations. The minimum was set at 10 documents per country and included 22 countries. Countries with the highest number of citations have darker spot. In the map, the UK, Benin, Francs and the USA had the darkest spots, i.e. highest citations

A total of 82 different countries and 1922 authors contributed to the publication of retrieved articles. Therefore, the average number of authors per article was 3.12. Of the 82 countries, there were 34 African countries and 12 Asian countries. The top ten productive countries were shown in Table 1. The United Kingdom (UK) ranked first followed by the United States of America (USA) and France. The productivity of countries in the top ten list was made mainly through international collaboration. The top list included seven African countries, one Asian, and three western countries. More than 90% of articles produced by the UK, France, Burkina Faso, Kenya, Cameron and Tanzania were produced by multiple authors from different countries, i.e. international collaboration. India has the least international collaboration followed by South Africa. VOSviewer network visualization map showed that the UK had collaborations mostly with Benin (relative link strength = 46) and the USA (relative link strength =37). For the USA, collaboration was mostly with the UK and Kenya (relative link strength = 17). For France, collaboration was mostly with the UK (relative link strength = 31) and Cote d’Ivoire (relative link strength = 21) (Fig. 2). Analysis of citation counts for countries with a minimum productivity of 10 documents showed that the UK had the largest citations followed by France, Benin, and the USA (Fig. 3).

**Table 1:** Top ten productive countries and international collaboration in malaria vector resistance to insecticides (1996–2015)

Rank		Publications^a^	%^a^	SCP	%	MCP	%
1^st^	UK	211	34.25	21	9.95	190	90.05
2^nd^	USA	119	19.32	23	19.33	96	80.67
3^rd^	France	95	15.42	6	6.32	89	93.68
4^th^	Benin	92	14.94	14	15.22	78	84.78
5^th^	South Africa	70	11.36	27	38.57	43	61.43
6^th^	India	54	8.77	50	92.59	4	7.41
7^th^	Cote d’Ivoire	42	6.82	5	11.90	37	88.10
8^th^	Burkina Faso	38	6.17	0	0.00	38	100.00
9^th^	Kenya	30	4.87	0	0.00	30	100.00
10^th^	Cameroon	29	4.71	1	3.45	28	96.55
10^th^	Tanzania	29	4.71	1	3.45	28	96.55

*Abbreviation*: *USA* United States of America, *UK* United Kingdom, *SCP* single country publications (intra-country collaboration), *MCP* multiple country publication (inter-country collaboration)

^a^Numbers do not add up to the total of 616 due to overall created by international collaboration

### Publications analysis based on institutions

A total of 1817 institutions/ organizations participated in the publication of retrieved articles. Therefore, an average of 2.95 different affiliations were present per article which is almost similar to the average number of authors per article. Most active institutions/ organizations involved in insecticide resistance are shown in Table 2. The most active institution/ organization was *Liverpool School of Tropical Medicine* followed by *London School of Hygiene & Tropical Medicine*. Half of top ten productive institutions were in Africa, two in the UK, one in France and one was WHO. Articles published by WHO and *center MURAZ*(Burkina Faso) had the highest percentage of highly cites articles. Four of the top active institutions/ organizations were academic institutions while the remaining where research centers or health organizations. The Center for Disease Control and Prevention (CDC) ranked 11^th^ with 21 publications. The *National Institute of Malaria Research in India* was the only Asian institution/ organization in the top active list. This institute had the lowest percentage of highly cited articles relative to those in the top active list.

**Table 2:** Top productive institutions/ organizations in malaria vector resistance to insecticides (1996–2015)

Rank	Institute	Affiliation country	Publications	%^a^ *N* = 616	HCA	%
1	*Liverpool School of Tropical Medicine*	UK	118	19.16	60	50.85
2	*London School of Hygiene & Tropical Medicine*	UK	73	11.85	40	54.79
3	*University of Witwatersrand*	South Africa	72	11.69	26	36.11
4	*IRD Centre de Montpellier*	France	66	10.71	33	50.00
5	*Centre de RechercheEntomologique de Cotonou*	Benin	60	9.74	21	35.00
6	*National Institute for Communicable Diseases*	South Africa	51	8.28	22	43.14
7	*University of Abomey-Calavi*	Benin	29	4.71	5	17.24
8	*National Institute of Malaria Research India*	India	25	4.06	4	16.00
9	*OrganisationMondiale de la Sante*	WHO	22	3.57	13	59.09
9	*Centre MURAZ*	Burkina Faso	22	3.57	13	59.09

^a^because of the overlap in publications, the percentages maybe greater than 100%

*Abbreviation*: *UK* United Kingdom, *USA* United States of America, *HCA* highly cited articles (had a citations ≥ 20)

### Publications analysis based on journals in which they were published

Retrieved articles were published in 134 different scientific peer reviewed journals. The journal that published most in this topic was *Malaria Journal* (*n* = 101; 16.40%) followed distantly by Parasites and Vectors journal (60, 9.74%). The top ten active journals in publishing articles on insecticide resistance are shown in Table 3. The total number of articles published by top ten journals was 333 articles equivalent to 54.06% of total retrieved articles. The total IF of articles published by top ten journals was 904.01, an average of 2.71 per article.

**Table 3:** Top ten productive journal in malaria vector resistance to insecticides (1996–2015)

Rank	Journal	Frequency	%	IF	TIF
1^st^	*Malaria Journal*	101	16.40	3.079	310.979
2^nd^	*Parasites And Vectors*	60	9.74	3.234	194.040
3^rd^	*Plos One*	44	7.14	3.540	155.760
4^th^	*Journal Of Medical Entomology*	25	4.06	1.712	42.800
4^th^	*Medical And Veterinary Entomology*	25	4.06	2.230	55.750
6^th^	*American Journal Of Tropical Medicine And Hygiene*	20	3.25	1.670	33.400
7^th^	*Transactions Of The Royal Society Of Tropical Medicine And Hygiene*	18	2.92	1.790	32.220
8^th^	*ActaTropica*	14	2.27	1.840	25.760
9^th^	*Insect Molecular Biology*	13	2.11	3.150	40.950
9^th^	*Journal Of The American Mosquito Control Association*	13	2.11	0.950	12.350
Total					904.009

*Abbreviation*: *IF* impact factor, *TIF* total impact factor

**Fig. 4:**
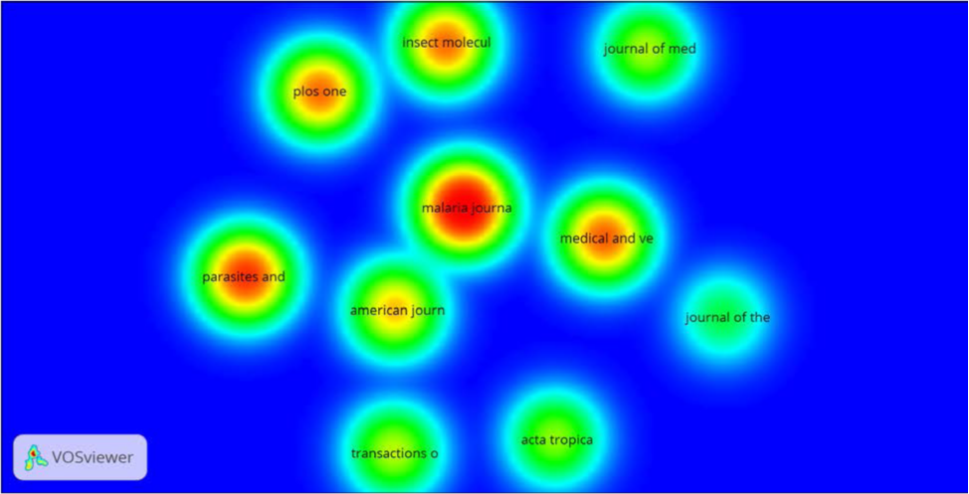
Density visualization map of citations for top journals in publishing articles on malaria vector resistance to insecticides (1996–2015)

**Fig. 5:**
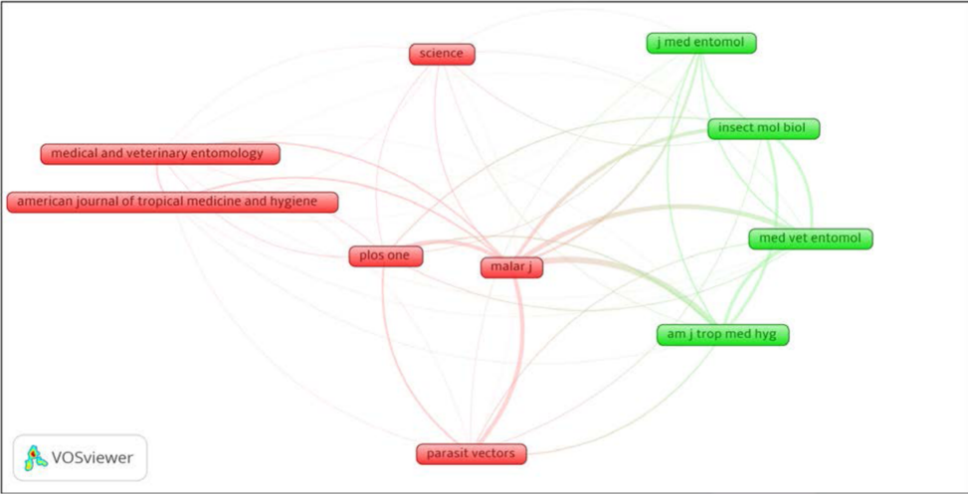
Network visualization map of co-citations for top journals in publishing articles on malaria vector resistance to insecticides (1996–2015)

VOSviewer density visualization of citation density of e top ten productive journals shows that *Malaria Journal* had the highest number of citations followed by *Parasites and Vectors* journal and *Medical and Veterinary Entomology* journal (Fig. 4). Co-citation analysis of journals with a minimum of 250 citation yielded 11 journals with the strongest link was between *Malaria Journal* and *American Journal of Tropical Medicine and Hygiene* (link strength = 2178) followed by the link between *Malaria Journal* and *Medical and veterinary Entomology* (link strength = 1717) (Fig. 5).

### Publications analysis based on the most active authors

**Fig. 6:**
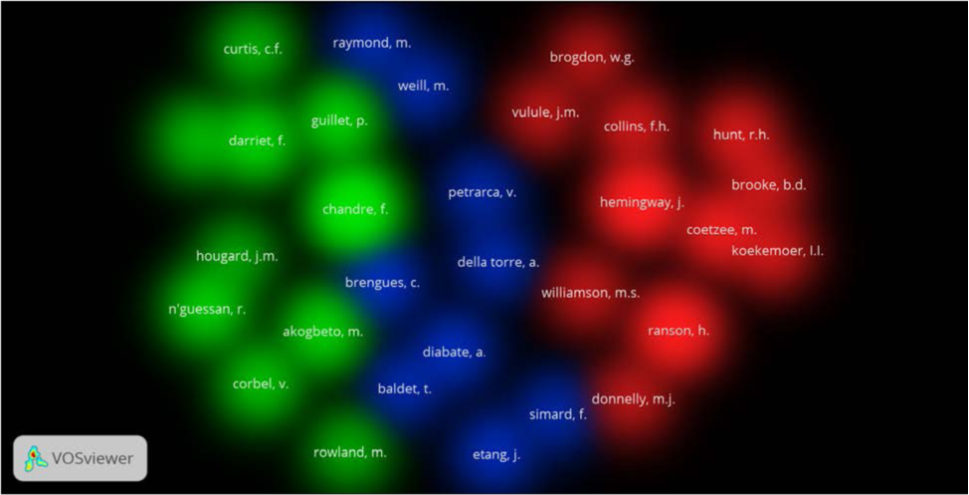
Density visualization map of author co-citation analysis. Authors within the same color cluster are co-cited together. Three clusters were obtained with red, blue and green colors. The map was created with a minimum of 250 citations per author which included 30 authors

**Fig. 7:**
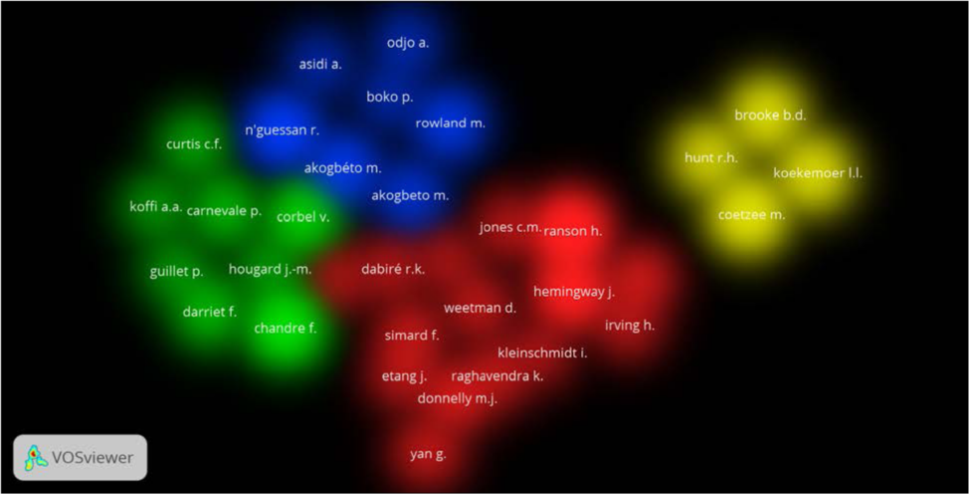
Density visualization map for author co-authorship analysis using a minimum of 11 documents per author which included 34 authors. Each color represents a group of authors with research collaboration

Authors active in insecticide resistance are shown in Table 4. The top active authors belonged to different countries and institutional affiliations. Four of the top active authors were from South Africa and there were from the UK. One author in the top list was from South East Asia, particularly from Thailand. VOSviewer co-authorship analysis (Fig. 6) showed that the top active authors existed in 4 different clusters seen in four different colors. The co-citation analysis showed that seven of the top active authors were usually co-cited together (Fig. 7).

**Table 4:** Top active authors in publishing articles on malaria vector resistance to insecticides (1996 – 2015)

Author	Frequency	%	Affiliation^a^	Co-citation^1^	Co-authorship^2^
Ranson, H.	63	10.23	Liverpool School of Tropical Medicine, Department of Vector Biology, Liverpool, United Kingdom	red	red
Akogbéto, M.	62	10.06	Centre de RechercheEntomologique de Cotonou, Cotonou, Benin	green	blue
Chandre, F.	49	7.95	IRD Institut de Recherche pour le Developpement, Paris, France	green	green
Coetzee, M.	49	7.95	National Institute for Communicable Diseases, Vector Control Reference Unit, Johannesburg, South Africa	red	Yellowish green
Hemingway, J.	49	7.95	Liverpool School of Tropical Medicine, Liverpool, United Kingdom	red	red
Brooke, B.D.	38	6.17	National Institute for Communicable Diseases, Centre for Opportunistic, Johannesburg, South Africa	red	Yellowish green
Koekemoer, L.L.	38	6.17	School of Pathology, Faculty of Health Sciences, Johannesburg, South Africa	red	Yellowish green
N’Guessan, R.^**3**^	38	3.41	Moshi, Pan-African Malaria Vector Research Consortium (PAMVERC), Tanzania OR London, UK	green	blue
Corbel, V.	32	5.19	Kasetsart University, Department of Entomology, Bangkok, Thailand	green	green
Hunt, R.H.	30	4.87	School of Pathology, Faculty of Health Sciences, Johannesburg, South Africa	red	red
Donnelly, M.J.	21	3.41	London School of Hygiene & Tropical Medicine, London, United Kingdom	red	Yellowish green

^a^Affiliation was obtained from author profile as shown in Scopus

^**● 1,** 2^ refer to the color of the cluster in Figs. 6 and 7 in which the authors are located

^**●** 3^ the author has 2 profiles in Scopus which were put together

### Publications analysis based on top cited articles

Top ten cited articles are shown in Table 5 [56–65]. At least four of the top ten cited articles were authored/ co-authored by Professor Ranson, H. Three of the top ten cited articles were published in *Insect Molecular Biology* journal. Six articles were about pyrethroid resistance and at least two were about DDT resistance. Pyrethroid Knockdown resistance (kdr), voltage-gated sodium channel gene, and voltage-gated sodium channel gene were main molecular biology topics in the top ten cited articles. The highest number of citations obtained was 444 for the article entitled “Molecular characterization of pyrethroid Knockdown resistance (kdr) in the major malaria vector *Anopheles gambiaes.s*.” [56].

**Table 5:** Top ten cited articles on malaria vector resistance to insecticides (1996–2015)

Rank	Author	Year	Title	Number of citations
1^st^	Martinez-Torres et al. [56]	1998	“Molecular characterization of pyrethroid knockdown resistance (kdr) in the major malaria vector Anopheles gambiaes.s.”	444
2^nd^	Hemingway et al. [57]	2004	“The molecular basis of insecticide resistance in mosquitoes”	357
3^rd^	Ranson et al. [58]	2011	“Pyrethroid resistance in African anopheline mosquitoes: What are the implications for malaria control?”	323
4^th^	Hargreaves et al. [59]	2000	“Anopheles funestus resistant to pyrethroid insecticides in South Africa”	318
5^th^	Ranson et al. [60]	2000	“Identification of a point mutation in the voltage-gated sodium channel gene of Kenyan Anopheles gambiae associated with resistance to DDT and pyrethroids”	291
6^th^	Ranson et al. [61]	2002	“Evolution of supergene families associated with insecticide resistance”	282
7^th^	Enayati et al. [62]	2005	“Insect glutathione transferases and insecticide resistance”	275
8^th^	N’Guessan et al. [63]	2007	“Reduced efficacy of insecticide-treated nets and indoor residual spraying for malaria control in pyrethroid resistance area, Benin”	256
9^th^	Chandre et al. [64]	1999	“Status of pyrethroid resistance in Anopheles gambiaesensulato”	222
10^th^	Ranson et al. [65]	2001	“Identification of a novel class of insect glutathione S-transferases involved in resistance to DDT in the malaria vector Anopheles gambiae”	215

## Discussion

### Resistance to insecticides

The objective of this study was to give a bibliometric overview of the resistance of the malaria vector to the approved insecticide. The timing of this study is important for at least three reasons: First, several international agencies are preparing strategic plans to control and eliminate malaria in the coming decade. Second, the resistance of malaria vector to pyrethroidis is escalating [66] and finally, 3) pyrethroid resistance will have serious implications on malaria vector control and thus malaria infection control [58]. Our study showed a noticeable increase in insecticide resistance in 2006. Several reasons could be cited to explain this rise, for example, the extensive use of pyrethroids in ITN, LLINs, and IRS in the past two decades is one reason [63, 67]. Furthermore, the extensive use of DDT and bendiocard in IRS contributed to this resistance. The utilization of insecticides in agriculture to control crop pests is also a potential reason for the emergence of malaria mosquito resistance [68].

### Resistance to pyrithroids

The high citations and h-index of retrieved articles on insecticide resistance is indicative of the importance of this issue [69, 70]. Malaria is one of the major global public health concerns and the control of malaria is a priority for many African countries. Such control cannot be achieved without controlling the vector. The use of insecticides in ITNs, LLINs and IRS was a success story and created an impressive reduction in malaria burden for countries in Africa and other continents [71]. The news for vector resistance attracted the attention of researchers, international organizations and health bodies and elicited a wave of publications regarding epidemiology, health consequences and molecular biology behind this resistance. Actually, the news on insecticide resistance reached a situation that needs an urgent action at all levels including research to maintain the success in malaria control [19]. Our study also showed that publications on pyrethroid resistance is a strong significant predictor of worldwide publication on malaria vector resistance. This is not surprising given that synthetic pyrethroids are the only WHO-recommended insecticides in treated nets [72]. The potential loss of pyrethroids as a weapon in fighting malaria vector was an alarming news to those involved in malaria control and initiated more extensive molecular research on this issue as well as research on developing effective and safe alternatives to pyrethroids in treated nets. Furthermore, in response to these alarming reports, the WHO developed the Global Plan for Insecticide Resistance Management in Malaria Vectors (GPIRM) [72].

### Active countries in research related to insecticide resistance of malaria vector

Our study showed that 34 African countries participated in the publications of retrieved articles on insecticide resistance. According to the WHO and since 2010, at least 60 countries have reported resistance to at least one class of insecticide and at least 49 countries have reported resistance to at least two or more different classes [73]. In Africa, resistance have been reported from many countries [24, 74–84]. Of particular importance are the African countries in which insecticide resistance has been shown to all four classes of insecticide. Such countries include Cote D’Ivoire and Mali [16, 85]. The involvement of the African countries in malaria research in general, and in insecticide resistance in particular, came largely through international collaboration as indicative of percentage of MCP from African countries listed in the top ten list. It is true that such countries have been suffering from malaria and other tropical diseases and it is a must to follow up in research on these disease, however, the limited expertise and resources of these countries necessitates the collaboration with other western developed countries particularly the UK and France given the historic relations between these countries and Africa. A description of benefits gained by collaboration was elaborated in an article discussing the China-Africa collaboration in malaria control and elimination [86]. Our study showed that four active institutions focusing on insecticide resistance were located in Africa, particularly South Africa, Benin and Burkina Faso. Of particular importance is the National Institute for Communicable Diseases (NICD) in South Africa which provides information on communicable diseases to support the government’s response to disease threats [73]. Although, the focus of vector resistance is directed toward Africa, other parts of the world in which malaria is an endemic disease had also participated in research and publications on this issue. Countries like India, China, Thailand and other Asian and South American countries have also contributed to the publications [16, 87–92]. Insecticide resistance in south eastern region and in Mekong area in particular has become of major threat given the reported resistance of *Plasmodium falciparum* to the most effective anti-malarial, artemisinin drugs [93, 94].

### Journals that published the most articles

Our study showed that *Malaria Journal* was the most active in publishing articles on vector resistance. It is the only journal that is dedicated for malaria research in the general sense. However, journals in tropical medicine, parasitology and medical entomology were also heavily involved in publishing work related to malaria vector insecticide resistance. The top active journals in malaria vector resistance had an IF suggesting that such topic is of an interest to editors of prestigious and strong journals. Of the top active journals, PLoS One journal is the only multidisciplinary journal that published articles of original research from all fields of science and medicine. The *Malaria Journal* played the central role in malaria research in general as shown in VOSviewer map where *Malaria Journal* had the largest number of citations compared to other journals is being commonly co-cited with almost all active journals. Again, regardless of the IF, the number of citations is indicative of the power and authority in the field of interest. The fact that malaria vector resistance is relatively a new topic, most of the retrieved documents were original research articles and less than 6% (34) of the retrieved documents were reviews, conference papers, editorials, letters and notes.

### Articles citation

Our study showed that articles on molecular mechanisms of malaria vector resistance are being mostly cited. This interest in the molecular biology aspects of insecticide resistance is most likely motivated by the search for new novel insecticides that are less prone to resistance. Point mutations in mosquitos described as Knockdown resistance (kdr) was extensively investigated to understand the molecular aspects of *Anopheles* mosquito resistance. Knockdown resistance (kdr) is the most common form of insecticide resistance and has been reported in many African and Asian countries [20, 81, 95–104]. One of the top cited articles also discussed the role of voltage gated sodium channels in insecticide resistance. The most common form of kdr is a mutation in voltage gated sodium channels in the central nervous system of the mosquito [102, 105–108]. Additionally, the role of glutathione transferases in conferring metabolic resistance was discussed in at least two of the top cited articles. Glutathione transferase is a form of metabolic detoxification in malaria vector. Such metabolic detoxification also includes alteration in P450 monooxygenases and esterases [109–113].

### Authors’ analysis

Our study showed that the most active authors were in South Africa in addition to authors in the UK, France and other countries. The top active authors in South Africa are being co-cited with active authors in the UK and VOSviewer showed that authors in South Africa and the UK have joint collaboration in this regard. South Africa is one of the African countries that had successfully controlled and confined malaria [114–118]. Actually, regional and international collaboration with African countries in malaria control programs had yielded positive results in malaria control [119].

This study is the first to discuss malaria vector resistance to insecticides. However, the authors acknowledge that this study has few limitations that need to be addressed. The fact that not all journal, particularly those issued from Africa or Asia, are indexed in Scopus made the number of retrieved documents less than the true accurate number. Furthermore, the authors did their best to use all keywords that are relevant to the topic and did a manual check for the retrieved articles, however a limited number of false positive or negative remain a possibility. With regard to the ranking, the authors extracted the information as is from Scopus. However, due to different spelling of authors or institutions, it is possible that some authors have multiple affiliations that the authors were not aware of and thus the ranking might not be 100% accurate. Finally, in VOSviewer, we always used a minimum number or threshold to draw the maps, thus not all the items are shown. Not being shown in the map does not mean that the item is not important or that the authors were biased toward any particular item. The authors were aware of all these limitations and did their best to minimize it to an acceptable level.

## Conclusion

Our study showed that publications on malaria vector resistance to insecticides is gaining momentum in the past decade. African countries and institutions had positive and noticeable contribution to publications in this topic. International collaborations with African countries in this field was also significant. Publications on this topic appeared in high impact journal and had high citations and h-index indicative of readability and quality. Molecular biology aspects of insecticide resistance were the most cited articles in the field. Authors from South Africa and the UK had noticeable collaboration and contribution to malaria vector resistance. Further research, collaboration, and funding is needed to investigate the epidemiology and molecular biology of this topic.

### Implications and future work

Studies investigating *anopheles* target-organ resistance is recommended, in addition to strategies to combat insecticide resistance must be kept a priority. Cross-resistance should be also investigated, as well as, the ecology of the target pest, novel biochemical targets, and new chemical approaches for pest control and the implications of utilizing more and newer insecticides.

## Abbreviations

WHO: World Health Organization
ITN: Insecticide treated nets
IRS: Indoor residual spraying
Kdr: Knockdown resistance
